# A Novel Microfluidic Chip for Fast, Sensitive Quantification of Plasma Extracellular Vesicles as Biomarkers in Patients With Osteosarcoma

**DOI:** 10.3389/fonc.2021.709255

**Published:** 2021-08-30

**Authors:** Yi-Qi Xu, Qi-Yuan Bao, Sai-Xi Yu, Qi Liu, Yan Xie, Xin Li, Yan-Jun Liu, Yu-Hui Shen

**Affiliations:** ^1^Department of Orthopedics, Ruijin Hospital, Shanghai Jiaotong University School of Medicine, Shanghai, China; ^2^Shanghai Institute of Cardiovascular Diseases, Shanghai Key Laboratory of Medical Epigenetics, International Co-laboratory of Medical Epigenetics and Metabolism (Ministry of Science and Technology), Institutes of Biomedical Sciences, Zhongshan Hospital, Fudan University, Shanghai, China; ^3^Engineering Research Center for Nanophotonics and Advanced Instrument, Joint Institute of Advanced Science and Technology, School of Physics and Electronic Science, East China Normal University, Shanghai, China

**Keywords:** ZnO-nanorods, microfluidic chip, vimentin, osteosarcoma, extracellular vesicles

## Abstract

Plasma circulating extracellular vesicle (EV) has emerged as a promising biomarker for diagnosis and prognosis of various epithelial tumors. However, fast and efficient capture of EVs with microfluidic chip in sarcoma remains to be established. Herein, we reported a ZnO-nanorods integrated (ZNI) microfluidic chip, where EV capture antibody was uniformly grafted to the surface of the ZnO-nanorods of the chip to enhance the plasma turbulence formation and the capture efficiency at the micro-scale. Based on osteosarcoma (OS) cell line, we demonstrated that a combination of CD81 and CD63 antibody on ZNI chip yielded the greatest amount of total EVs, with an extra sensitive limit of detection (LOD) of ~10^4^ particles mL^-1^. Furthermore, the addition of fluorescent labeling of Vimentin (VIM), a previously reported sarcoma cell surface biomarker, could enabled the dual visualization of total plasma EVs and VIM-positive EVs from OS patients’ plasma. Based on our ZNI chip, we found that the amount of plasma total EVs was significantly different between OS and healthy donors (1562 a.u. *versus* 639 a.u., p< 0.05), but not between metastatic and nonmetastatic OS (p> 0.05). Interestingly, patients with metastatic disease had a significantly greater amount of VIM-positive EVs (1411 a.u. *versus* 231 a.u.., p< 0.05) and increased VIM-positive/total EVs ratio (0.943 *versus* 0.211, p< 0.05) in comparison with the nonmetastatic counterpart. Therefore, our ZNI microfluidic chip has great potential for the fast quantification of plasma EVs, and the microfluidic-based quantification of total and VIM-positive EVs might serve as a promising biomarker for the diagnosis and surveillance in OS patients.

## Introduction

Liquid biopsy of circulating extracellular vesicle (EV) has nowadays gained an increasing popularity as a promising source of prognostic and therapeutic biomarkers for several common types of epithelial cancer (1–3). Tumor-derived EV has been implicated in multiple steps of cancer pathogenesis and metastasis, including the organotropic determination, microenvironment formation, immune response (4–7). Unlike other liquid-based approach such as circulating tumor cell (CTC) and cell-free tumor DNA (ctDNA), EV is reported to be actively secreted and functionally transported within blood stream, with a favorable stability and high abundance (~10^10^ particles/mL) (8–10), thereby regarded as a promising source of cancer biomarker.

For sarcoma, however, capturing and quantifying of circulating EVs with fast and simple tools remains to be exploited. Currently, the isolation of EVs mainly relies on ultracentrifugation (UC), which is limited by large sample consumption, low sample recovery rate and complicated procedures (11–14). Other methods such as ultra-filtration and precipitation-based commercial kits suffer from poor standardization and the contamination of non-vesicular particles (15–18). In recent years, microfluidic chips with nanostructure have been increasingly accepted as a promising tool for the separation and detection of EVs in a high-throughput and integrable manner (19–23). Nanomaterials have strong adsorption capacity of EVs owing to their high surface area to volume ratio and reduced near-surface flow stagnation, thereby enhancing the combination of vesicles and the surface of nanomaterials (3, 24, 25). Among the many nanomaterials, ZnO-nanorods are especially advantageous in biological detection due to their low production cost, easy preparation, robustness, and biocompatibility (26–28). Furthermore, large specific surface area, interface electron transfer effect and optical waveguide effect of ZnO-nanorods could significantly enhance the fluorescence signal (29–31).

**Figure 1:**
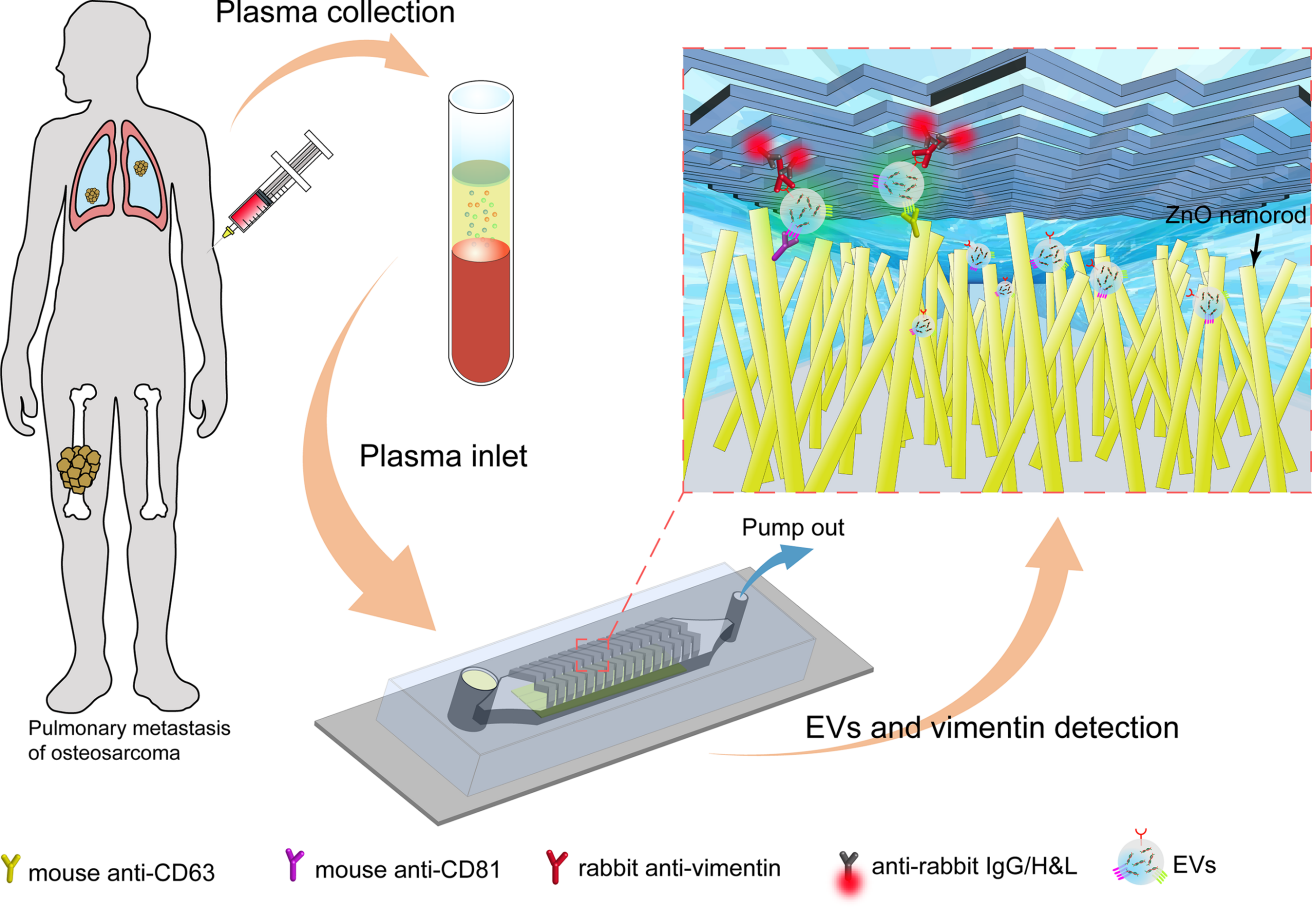
The overall process flow of the ZnO nanorods integrated (ZNI) microfluidic chip for detection of EVs from osteosarcoma patients. First, the peripheral blood of osteosarcoma patients was collected, and the blood cells and plasma were separated by centrifugation. At the other end of the chip, a syringe pump was used to pump the plasma in the sample reservoir at a fixed speed to capture EVs in the plasma. Next, the lipophilic membrane dye 3,3’-dioctadecyloxacarbocyanine (DiO) was used to quantify the captured EVs in the chip. Finally, EV membrane vimentin (VIM, a sarcoma biomarker) was detected using a second signal based on fluorescence conjugated antibodies.

Herein, we designed a ZnO-nanorods integrated (ZNI) microfluidic chip (**Figure 1**). Under the synergistic effect of the herringbone (HB) structure and ZnO nanorods, the formation of turbulence of plasma flowing through the chip increased the collision probability of EVs with nanorods, resulting in a favorable capture efficiency at the micro-scale (22, 32, 33). Next, we performed a series of optimization and validation study using EVs from cell line and patient plasma of osteosarcoma, the most common bone sarcoma. We found that our device had an extremely low limit of detection (LOD) of ~10^4^ particles/mL, which was drastically below the EV concentration in plasma. Furthermore, our microfluidic-based EV quantification could not only distinguish osteosarcoma patients from healthy donors, but also the metastatic diseases from the non-metastatic counterparts.

## Methods and Materials

### Preparation of Microfluidic Device

**Figure 2:**
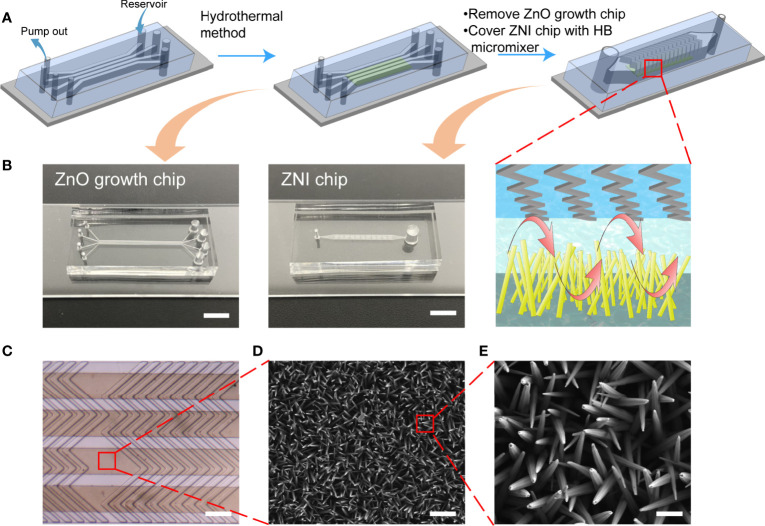
The flow chart of manufacturing the ZNI chip. **(A)** The ZnO nanoarray was prepared in ZnO growth chip by a hydrothermal method. 4 groups of nanoarrays were prepared simultaneously. Then, the ZnO growth chip was peeled off and bonded the chip with the herringbone (HB) micromixer structure to the top of the nanoarray to complete the assembly of the device. **(B)** Photographic image of the ZnO growth chip and ZNI chip. Scale bar: 4mm. **(C)** Stereoscope image of ZNI chip. Scale bar: 200 μm. **(D**, **E)** Scanning electron microscope (SEM) micrographs of ZnO nanorods. **(D)** Scale bar: 1μm. **(E)** Scale bar: 200nm.

The ZnO growth chip and the ZNI chip were fabricated using a standard soft lithography process. The SU-8 2050 photoresist (MicroChem) was spin-coated on the silicon wafer. Following ultraviolet (UV) exposure and development, the mold was treated with trimethylchlorosilane and then filled with PDMS (RTV615) prepolymer at a 10:1 ratio of base polymer to cross-linker. Next, the mold filled with PDMS prepolymer was de-bubbled in a vacuum manner. The PDMS replicas were peeled off after curing at 80°C for 2 hours. The ZnO growth chip consisted of 4 channels with a width of 200 μm and a height of 75 μm, and the ZNI chip consisted of the main microfluidic channel (height: 10, 20, 30, 40 μm) and the HB structure (height: 5 μm) (**Figure 2B**).

### Preparation and Functionalization of ZnO-Nanorods on ZNI Chip

The seed solution, 10 mM zinc acetate ethanol solution, was spin-coated on the clean glass slide at a rotation speed of 2000 r/min, and spin-coating was repeated three times. The glass slide was placed on a heating platform at 300°C for 2 h to complete the preparation of seed layer. Then, 0.2g polyethyleneimine (PEI) (12.5 mM), 0.372g Zn (NO_3_)_2_·6H_2_O (25 mM), 0.0875g hexamethylenetetramine (HMTA) (12.50 mM) were dissolved in deionized water in a 50 mL volumetric flask to prepare ZnO growth solution, and NH_3_·H_2_O was added to adjust the pH to 10.60. The ZnO growth chip was bonded to the glass slide covered with the seed layer, the growth solution was added to the reservoir, and the syringe pump was used to pump the growth solution at a speed of 4.8 μL/min. ZnO nanorods were prepared by local heating at a temperature of 90°C for 3 h, which was the optimized parameter for acquiring the largest binging surface area of protein accordingly to our previous work (30). Then, the ZnO growth chip was peeled off and the ZNI chip with HB structure was bonded to the glass slide with ZnO-nanorods to complete the final device assembly (**Figures 2A, C**). The morphology of ZnO-nanorods was characterized by scanning electron microscopy (ZEISS Supra 40VP SEM) (**Figures 2D, E**).

To functionalize the nanorods with EV capture antibody, the surface of ZnO-nanorods by exposure to 4% (3-mercaptopropyl) trimethoxysilane (MPS) anhydrous ethanol solution at room temperature for 30 min. Excess silane was washed away with anhydrous ethanol. Next, 0.25mM N-maleimidobutyryl-oxysuccinimide ester (GMBS) DMSO solution was injected into the ZNI chip to modify the surface of nanorods for 40 min. After washing with PBS, 10 μg/mL Protein G PBS solution was fed into the chip to coat the surface for 1 h at 4°C (34). After washing with PBS, 10 μg/mL anti-CD63 and anti-CD81 mixed antibody PBS solution was pumped into the chip channel for 1 h at 4 °C. At the end, the chip was blocked with 3% BSA for 30 min and stored at 4°C before the experiments (**Figure S1**). Fourier Transform infrared spectroscopy (FT-IR) was used to detect the relevant chemical groups in each step of the antibody covalent coupling (**Figure S2**). The CD63+CD81 double-antibody was characterized by fluorescently labeled goat anti-mouse IgG (**Figure S3**). The information of the materials and reagents used in this research were displayed in **Table S1**.

### Collection of Plasma EVs

The study protocol was approved by the ethical committee of the institution according to relevant guidelines and was prospectively collected in our longitudinal observational clinical research project registered at clinicaltrial.gov (trial ID: NCT03108677). OS patients were free of any systemic treatment for at least 1 month before the blood draw to minimize the potential intra-individual variation. All plasma was collected using Vacutainer Glass Blood Collection Tubes with Acid Citrate Dextrose (BD, USA) and centrifuged at 2000*g* and 4000*g* respectively for 10 min to remove cell debris before introduced into the ZNI chip platform. For the characterization of plasma EVs, the plasma was then diluted 1:2 with PBS and filtered with the 0.45μm filter to remove the larger particles. Following ultracentrifugation at 110,000*g* for 11 hours and another ultracentrifugation at 110,000*g* for 70 minutes, the final EV pellets were resuspended in PBS and performed nanoparticle tracking analysis (NTA) as validation for quantification of plasma EVs.

### Isolation of OS Cell Line-Derived EVs

OS cell lines were purchased from The Cell Bank of Type Culture Collection of Chinese Academy of Sciences, Shanghai, China (www.cellbank.org.cn) with corresponding STR profiling as cell-line authentication. The medium we used was EV-depleted complete medium (EDCM) consisted of: DMEM+10% EV-depleted FBS+1% P-S.45 mL of culture supernatant from osteosarcoma cell lines HOS, 143B, U2OS, and MG63 was centrifuged at 2,000*g* for 15 min under 4°C to remove cell debris. The obtained supernatant was then centrifuged at 10,000*g* for 30 min under 4°C and filtering with the 0.45μm filter to remove the remaining cell debris and microvesicles. Next, the supernatant was centrifuged at 100,000*g* for 90 min, and the precipitate was resuspended in PBS followed by another ultracentrifugation at 100,000*g* for 90 min under 4°C. Finally, the precipitate was resuspended in PBS and stored at -20°C for future western blot analysis.

### Characterization of EVs

The morphology of the HOS-derived EVs was characterized using a Tecnai G2 20 TWIN transmission electron microscope (TEM). 2 µL of EV pellet was loaded on a 400-mesh carbon-coated copper grid and then negatively stained with 2% phosphotungstic acid for 10 min. After removal of the excess dyes, the prepared sample was left to dry at room temperature and observed under a voltage of 200 kV. For characterization of the captured EVs, the EVs immobilized on ZnO nanorods were fixed in 4% paraformaldehyde (PFA) for 1 h. The samples were dehydrated by sequential immersion in 30, 50, 75, 85, 95, and 100% ethanol solutions for 10 min per solution. After overnight lyophilization, sputter-coating with gold was performed at room temperature. The morphology of EVs immobilized on ZnO nanorods was then observed using SEM. To quantify the EV amount and size distribution, isolated EVs were proceeded with nanoparticle tracking analysis (NTA) as a gold standard. The videos of 60-sec duration taken by its camera 0.743 µm/px are analyzed with the Software (ZetaView 8.04.02).

### EV Capture and Quantification Using ZNI Chip

100 μl HOS-derived EV suspension, which was used to optimize the functional parameters, was pumped through the ZNI chip coated with anti-CD63 and CD81 antibodies at a flow rate of 2 μL/min using a micro syringe pump, and then 50 μM DiO membrane dye was injected into the chip at a speed of 2 μL/min and incubated at room temperature for 30 min. After being rinsed three times with PBS, fluorescence microscope (Nikon Ti2-U) was used to complete the quantification of EVs. Besides, 50 μL plasma (PBS diluted to 200 μL) was introduced into the ZNI chip coated with anti-CD63 and CD81 antibodies *via* the same procedure as above to complete DiO labeling of EVs. After being rinsed three times with PBS, 10 μg/mL rabbit anti-vimentin (VIM) antibody was pump into the chip at a speed of 1 μL/min for 40 min. The chip was rinsed 3 times to wash away excess antibody, 20 μg/mL Alexa Fluor 647 labeled goat anti-rabbit IgG (H+L) was pump into the chip at a speed of 1 μL/min for 40 min and then washed 3 times with PBS. Finally, the fluorescence microscope (Nikon Ti2-U) was used for observation and image J software was used to quantify the fluorescence signal collected on the chip. When the fluorescence signal data was processed, we used the relative fluorescence intensity I. I=(I_a_-I_b_)/t. I_a_ represents the absolute fluorescence signal on the image, I_b_ represents the background fluorescence signal, and t represents exposure time. When analyzing the data, we used the GraphPad Prism 8 software to perform the Mann–Whitney U test, where a P value of less than 0.05 is considered statistically significant. The result of ultracentrifugation followed by NTA (current golden standard) and that of the ZNI chip (without the need of ultracentrifugation) were compared to verify the accuracy of EV quantification of our device.

### Western Blot Analysis of EVs

The EVs derived from HOS, 143B, U2OS, and MG63 cells were mixed with RIPA to lyse the vesicles and extract the protein. 20 μL of each sample was added to a 10% SDS-PAGE Bis-Tris gel, and then performed electrophoretic separation. Afterwards, the protein on the gel was transferred to the nitrocellulose membrane, and then blocked with 5% BSA on a shaker at room temperature for 1 h and washed with TBST 3 times. Anti-CD9 (Abcam), anti-CD81 (Abcam), anti-CD63 (Abcam) and anti-VIM (CST) antibodies were added and incubated for 11 h on a shaker at 4°C. After being washed 3 times, secondary antibody was added and incubated for 1.5 h at room temperature. After being washed, the nitrocellulose membrane was exposed and finally completed protein qualitative detection.

### Validation of VIM Expression in Public Database

To validate VIM expression as a potential biomarker of metastatic OS, we retrieved the data of 127 OS patients (the largest dataset in the database) regarding the association of gene expression with the likehood of metastasis from R2 database (35). VIM expression was dichotomized into VIM-high expression and VIM-low expression based on its median value. Kaplan-Meier survival analysis was performed to validate VIM as a biomarker of OS metastasis.

## Results

### Fabrication and Characterization of the ZNI Chip

**Figure 3:**
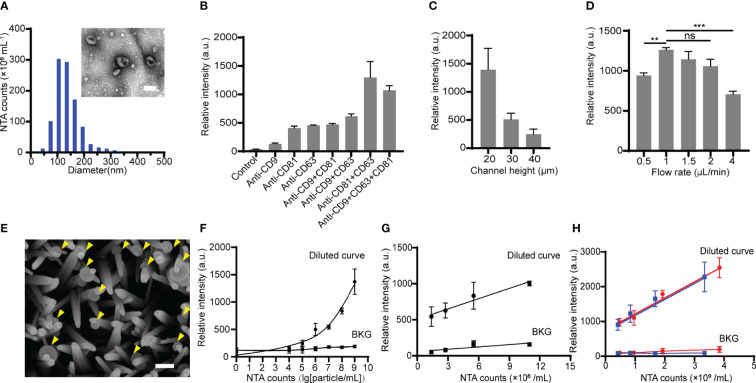
Optimization of the ZNI chip for EV capture. **(A)**Transmission electron microscope photograph and nanoparticle tracking analysis (NTA) of HOS-derived EVs. Scale bar: 100nm. **(B)** The effect of various capture antibody combination modes on HOS-derived EV detection efficacy. **(C)** The effect of various height of the main microfluidic channel of ZNI chip on HOS-derived EV detection efficacy. **(D)** The effect of various flow velocities ranging from 0.5 μL/min to 4 μL/min on HOS-derived EV detection efficacy. **(E)** Scanning electron microscope photographs of HOS-derived EVs captured by ZnO nanorods. Scale bar: 150nm. **(F)** Calibration curves for quantifying total HOS-derived EVs using ZNI chip under optimized function parameters. **(G)** Correlation of the EVs level between NTA and HOS-derived EVs resuspended in EV-free plasma. **(H)** Correlation of the EV level between NTA and patient-derived EVs resuspended in EV-deprived plasma using ZNI chip under optimized function parameters. BKG: background. Error bars correspond to the standard deviation (n = 3). ns: non-significant, p > 0.05, **p< 0.01, ***p < 0.001. “Two-way repeated-measures ANOVA” statistical analysis method was used to appraise the significance of different parameters as well as the batch effect in each experiment of **(B–D)**.

The ZnO growth chip, consisting of 4 channels with a width of 200 μm, a height of 75 μm, and a length of 2 cm was first reversibly bonded to a glass slide spin-coated with a ZnO seed layer and then covered with herringbone (HB) micromixer (**Figures 2A, B**) to produce turbulent flow and minimize the laminar flow in the microchannel. Under the parallel HB micro-structure (**Figure 2C**), the hexagonal prism structure of zinc oxide wurtzite left an interspace of 10~100 nm between nanowires (**Figures 2D, E**), exerting a size exclusion-like effect for retaining EVs (**Figure 3E**) (36, 37).

### Optimization of the ZNI Chip for EV Capture

EVs derived from HOS, a commonly used osteosarcoma cell line, as well as 3 replicates of microfluidic chip studies (manufactured in 3 batches) were performed for further optimizing the parameters of the ZNI chip. As expected, HOS-derived EVs exhibited cup- or spherical-shaped morphologies ranging from 30 to 200 nm (**Figure 3A**). Among various single or combination of capture antibodies, there is a significant difference in the quantity of the captured EV among each parameter (F= 16.62, p< 0.05). The quantity of the total captured EVs was found to be highest when CD63+CD81 double antibody was used (**Figure 3B**). Furthermore, the micro-channel of ZNI chip was frequently clogged at the height of 10 μm, and the greatest amount of EVs could be captured at the channel height of 20 μm (**Figure 3C**). Similarly, we observed a significant effect of the channel height to the capture result (F= 20.05, p< 0.05). Next, various injection flow rates ranging from 0.5 μL/min to 4 μL/min (**Figure 3D**) were further optimized. Interestingly, the capture efficiency reached a plateau from 1 μL/min~2 μL/min, and then drastically dropped at the flow rate of 4 μL/min in comparison to other flow rates. The flow rate was also found to significantly affect the EV capture quantity (F= 11.24, p< 0.05). Considering the experimental efficiency, we finalized 2 μL/min as the optimal injection flow rate for the subsequent clinical sample detection.

Using repeated measures ANOVA, we found no statistical significance of the batch effect among the three replications (p>0.05), suggesting that EV quantification using our microfluidic device was generally reproducible.

### Microfluidic-Based Quantification of Cell Line and Plasma EVs

With the aforementioned parameters of ZNI chip, the captured HOS-derived EVs were observed to be densely bonded to ZnO-nanorods (**Figure 3E**). A calibration curve between the fluorescence intensity and the logarithm of the EV concentration from 10^4^ particles/mL to 10^9^ particles/mL was obtained, with the limit of detection (LOD) being ~10^4^ particles/mL in comparison with background signal (ZNI chip without capture antibodies) (**Figure 3F**). To exclude the potential confounding effect of blood plasma to microfluidic quantification of EVs, HOS- as well as plasma-derived EV resuspension of known concentration was diluted into EV-free plasma into various concentration. Remarkably, we found a robust correlation of the NTA results with the microfluidic EV signal (R^2^=0.982 p=0.009), but not background signal (ZNI chip without capture antibodies) (R^2^=0.725 p=0.476) (**Figures 3G, H**), highlighting the quantification accuracy of our device despite the context of plasma as a complex bio-fluid.

### EV Quantification in OS Patients and Healthy Donors

**Figure 4:**
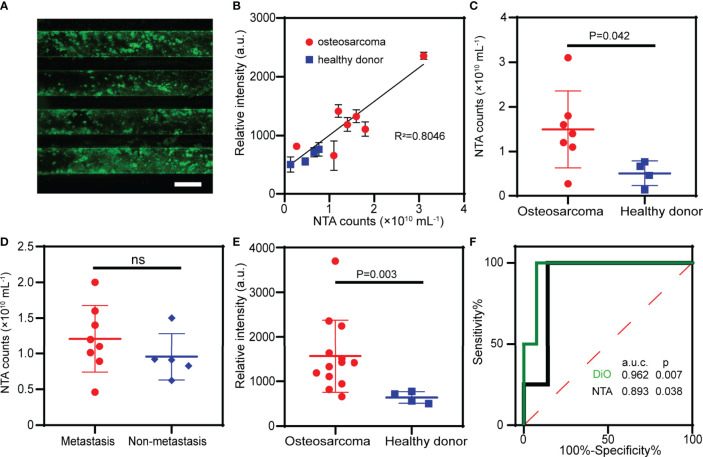
Clinical validation of the ZNI chip in patients of osteosarcoma (OS). **(A)** Fluorescence image of EVs from plasma captured by ZNI chip. Scale bar: 200μm. **(B)** Correlation analysis of NTA and ZNI chip fluorescence quantification of plasma EVs in 7 OS patients and 4 healthy donors. **(C)** NTA of the plasma EVs from OS patients and healthy donors. **(D)** NTA of the plasma EVs from OS patients with metastasis and without metastasis. **(E)** Fluorescent quantitation of plasma EVs from OS patients and healthy donors. **(F)** Receiver operating characteristic (ROC) analysis of plasma EVs quantified by ZNI chip and NTA between OS patients and healthy donors. The statistical difference of the two groups was compared using the Mann-Whitney U test. ns, non-significant.

In order to evaluate the clinical utility of the ZNI chip as a fast screening tool of liquid biopsy, blood samples from OS patients (*n*=13) and healthy donors (*n*=4) (**Table 1**) were used to quantify the total EVs in plasma (**Figure 4A**). Of the 13 OS samples, 7 were also measured by NTA as a validation. As expected, the fluorescence signal measured on the ZNI chip was highly correlated with NTA results (R^2^=0.805 p<0.05) (**Figure 4B** and **Figure S4**). NTA demonstrated that the extracellular vesicle concentration was significantly different between OS patients and the healthy control (1.5×10^10^ particles/mL *vs* 5.1×10^9^ particles/mL, p=0.042) (**Figure 4C**), but not between metastasis and non-metastasis patents (1.3×10^10^ particles/mL *vs* 1.0×10^10^ particles/mL, p > 0.050) (**Figure 4D**). Consistently, based our ZNI chip, the total EVs were significantly higher in OS patents than the healthy control (1562 a.u. vs 639 a.u.., p= 0.003) (**Figure 4E**). The Receiver Operating Characteristic (ROC) curve suggests that our ZNI chip has a favorable diagnostic ability, with an area under curve (AUC) of 0.962 (**Figure 4F**).

**Table 1:** Clinical information of osteosarcoma (OS) patients and healthy donors (HD) enrolled in this study.

Patient No.	Gender	Age (year)	Histological Subtype	Clinical Stage	Prognosis
P01	Female	11	Conventional OS	IIIB	Metastasis
P02	Male	62	Conventional OS	IIIB	Metastasis
P03	Male	8	Conventional OS	IIIA	Metastasis
P04	Male	20	Conventional OS	IIIA	Metastasis
P05	Female	19	Periosteal OS	IIIB	Metastasis
P06	Male	8	Telangiectatic OS	IIIB	Metastasis
P07	Male	25	Conventional OS	IIIB	Metastasis
P08	Female	9	Conventional OS	IIIB	Metastasis
P09	Male	15	Conventional OS	IB	Non-metastasis
P10	Male	17	Conventional OS	IA	Non-metastasis
P11	Male	14	Conventional OS	IB	Non-metastasis
P12	Male	13	Conventional OS	IB	Non-metastasis
P13	Female	17	Conventional OS	IIA	Non-metastasis
HD01	Male	25	N/A	N/A	N/A
HD02	Male	24	N/A	N/A	N/A
HD03	Male	25	N/A	N/A	N/A
HD04	Male	26	N/A	N/A	N/A

### Detection of VIM Positive EVs as a Potential Metastatic Biomarker of OS

**Figure 5:**
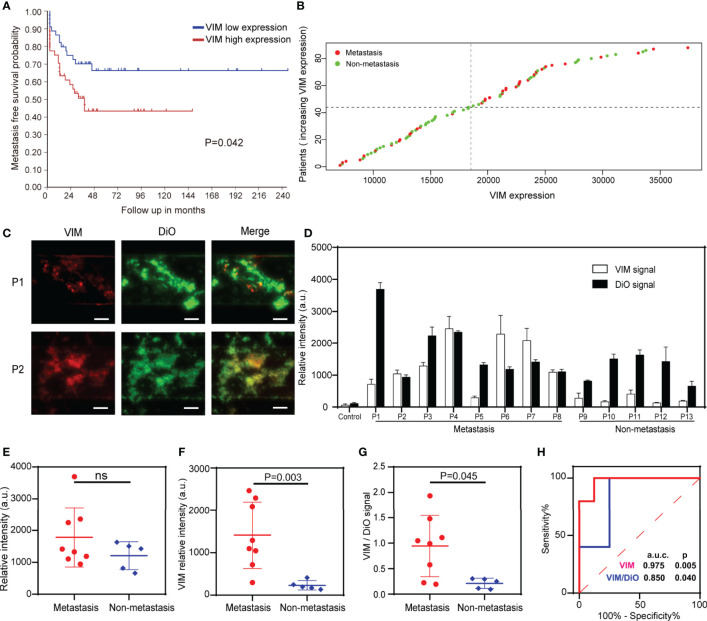
Detection of total EVs and VIM-positive EVs for the surveillance of OS metastasis. **(A)** Metastasis-free survival probability curve of OS patients with VIM high expression and low expression. **(B)** Scatter diagram of VIM expression of OS patients with and without metastasis. **(C)** Exemplary images of immunofluorescence of total EVs and VIM-positive EVs in 2 plasma samples. Scale bar: 100 μm. **(D)** Quantification of the EV membrane VIM directly from the plasma of the metastatic OS patients (*n* = 8) and non-metastatic patients (*n* = 5). Error bars indicate s.d. (*n* = 3). **(E)** Fluorescence quantification of the total EVs in OS patients with and without metastasis. ns, non-significant. **(F, G)** The VIM fluorescence signal and the VIM/DiO fluorescence ratio of the plasma EVs derived from OS patients with and without pulmonary metastasis. The statistical difference of the two groups was compared using the Mann-Whitney U test. **(H)** ROC analysis of VIM absolute signal and VIM/DiO ratio between the metastasis group and the non-metastasis group.

To date, there is no liquid biopsy-based biomarker for the metastasis of OS. We, therefore, sought to investigate the potential utility of a previously reported sarcoma CTC surface marker – VIM (38) - on the captured EVs in our microfluidic chip as a metastatic biomarker of OS. VIM was found to be expressed on the EVs from common OS cell lines such as HOS, 143B, MG63 and U2OS (**Figure S6**), and the upregulation of VIM in tumor sample was associated with the patients’ metastasis-free survival for 127 OS patients in R2 database (35) (**Figures 5A, B**). We, therefore, quantify the VIM expression status on the captured EVs in our device. Surprisingly, we observed fluorescent co-localization of the VIM (red) with total EVs (green) on the ZNI chip (**Figures 5C** and **S5**), while adding DiO/Alexa Fluor 647 to ZNI chip without EV capture antibodies yielding no or minimal fluorescence (negative control), excluding the possibility of nonspecific adsorption of fluorescent dyes (**Figure S7**). Furthermore, the fluorescent intensity of VIM and total EVs tremendously varied among OS patients (**Figure 5D**). Although there was no significant difference in total EVs between metastasis and non-metastasis subgroup (**Figure 5E**), the fluorescent signal of VIM as well as VIM/total EVs ratio were significantly higher in the metastasis compared to the non-metastatic group (**Figures 5F, G**). The ROC analysis suggested that VIM and VIM/DiO ratio could be a diagnostic biomarker for OS metastasis (**Figure 5H**). The statistical data of VIM and DiO relative fluorescent intensity from every specimen of this research is displayed in the **Supplementary Table**.

## Discussion

To our knowledge, there were no previous studies focusing on the optimization and validation of detecting OS-derived EVs using a microfluidic approach. We, for the first time, demonstrated a microfluidic chip integrated with ZnO nanorods, which could fast and effectively separate and quantify EVs in the plasma sample of OS patients. By optimizing the functioning parameters based on OS-derived EVs, we have further improved the detection limit to as low as 1.1×10^4^ particles/mL, which is drastically lower than previously reported (26), and the plasma concentration of EV (9). Excellent reproducibility of ZNI chip reveals the immense potential of clinical translation.

Furthermore, our device exceeded previous microfluidic-based isolation techniques in several aspects. Microfluidic chips based on size filtration, acoustic field, and electric field have been widely used in the separation and purification of EVs. Dong et al. developed an ExoID-Chip, which can achieve high-efficiency separation and sensitive detection of EVs through filtration (1). Wu et al. developed an acoustofluidic platform for EVs (39). In addition, Ibsen et al. used alternating current electrokinetic microarray chips to quickly extract EVs from human plasma (40). However, the EVs obtained by these methods were contaminated with non-vesicular particles, which affected subsequent biological analysis (15). Therefore, the isolation method based on immunoaffinity is more reasonable. Chen et al. presented a ZnO nanowire chip for immunocapture and colorimetric detection of EVs. However, the chip manufacturing time is long (>12 h) and the detection process is complicated (26). Our ZNI chip not only greatly reduces the manufacturing time (<5h), but also has simple detection steps and greatly improves the limit of detection (1.1×10^4^ particles/mL).

Currently, the liquid biopsy for the surveillance of tumor recurrence in OS remains still lacking. Through the analysis of clinical samples, our report was the first one to exploit the possibility of quantifying total extracellular vesicle to distinguish OS patients from healthy controls and identifying OS patients with metastasis from those without based on EV membrane VIM expression. Remarkably, our device could simultaneously quantify EVs and EV membrane biomarker in one single run, which only requires 50 μL of archived or fresh plasma samples. In addition, unlike traditional ultracentrifugation as a time-consuming process, it only took about 2 hours from sample collection to signal acquisition. Therefore, our ZNI chip could serve as a portable and efficient tool of liquid biopsy for total EV isolation and high-sensitivity biomarker detection for OS and could be easily adapted for EV membrane biomarker detection for other malignancies. The accuracy of our device might be further improved by introducing a standard sample with a known EV concentration as a control for minimizing the inter-assay variability for further applications. In general, our study suggests that the microfluidic detection of plasma EV membrane biomarker is a promising liquid biopsy technique in the diagnosis and treatment of OS.

## Data Availability Statement

The raw data supporting the conclusions of this article will be made available by the authors, without undue reservation.

## Ethics Statement

The studies involving human participants were reviewed and approved by Ruijin Hospital Ethics Committee. Written informed consent to participate in this study was provided by the participants’ legal guardian/next of kin.

## Author Contributions

Y-QX: Conceptualization, Methodology, Investigation, Writing - original draft. Q-YB: Methodology, Writing - original draft. S-XY: Investigation. QL: Investigation. YX: Conceptualization. XL: Conceptualization. Y-JL: Conceptualization, Methodology, Writing - review and editing, Supervision, Funding acquisition. Y-HS: Conceptualization, Methodology, Writing - review and editing, Supervision, Funding acquisition. All authors contributed to the article and approved the submitted version.

## Funding

This work was supported by the National Natural Science Foundation of China (NSFC, Grant Nos. 81773298 and 31870978); Shanghai Science and Technology Committee (Grant No. 17411951900) and Clinical Research Plan of SHDC (Grant No. SHDC2020CR3078B).

## Conflict of Interest

The authors declare that the research was conducted in the absence of any commercial or financial relationships that could be construed as a potential conflict of interest.

## Publisher’s Note

All claims expressed in this article are solely those of the authors and do not necessarily represent those of their affiliated organizations, or those of the publisher, the editors and the reviewers. Any product that may be evaluated in this article, or claim that may be made by its manufacturer, is not guaranteed or endorsed by the publisher.
