# Supplementary material for: A Novel Microfluidic Chip for Fast, Sensitive Quantification of Plasma Extracellular Vesicles as Biomarkers in Patients With Osteosarcoma
Source: Front Oncol. 2021 Aug 30;11:709255. doi: 10.3389/fonc.2021.709255 (PMC8437394; doi:10.3389/fonc.2021.709255)
Supplement: Supplementary file 1 [file DataSheet_1.docx]

**Supplementary Material**

**Contents**

S1. The scheme of chemical modification of ZnO nanorods surface 2

S2. Fourier-transform infrared spectroscopy (FT-IR) spectra 3

S3. Characterization of the antibodies on the surface of ZnO-nanorods 4

S4. Nanoparticle tracking analysis (NTA) of clinical samples 5

S5. The co-fluorescence on the ZNI chip 6

S6. Western blot analysis of extracellular vesicles from osteosarcoma cell line 7

S7. Negative controls of DiO and VIM fluorescence 8

Table S1. The materials used in this research. 9

**S1. The scheme of chemical modification of ZnO nanorods surface**

**
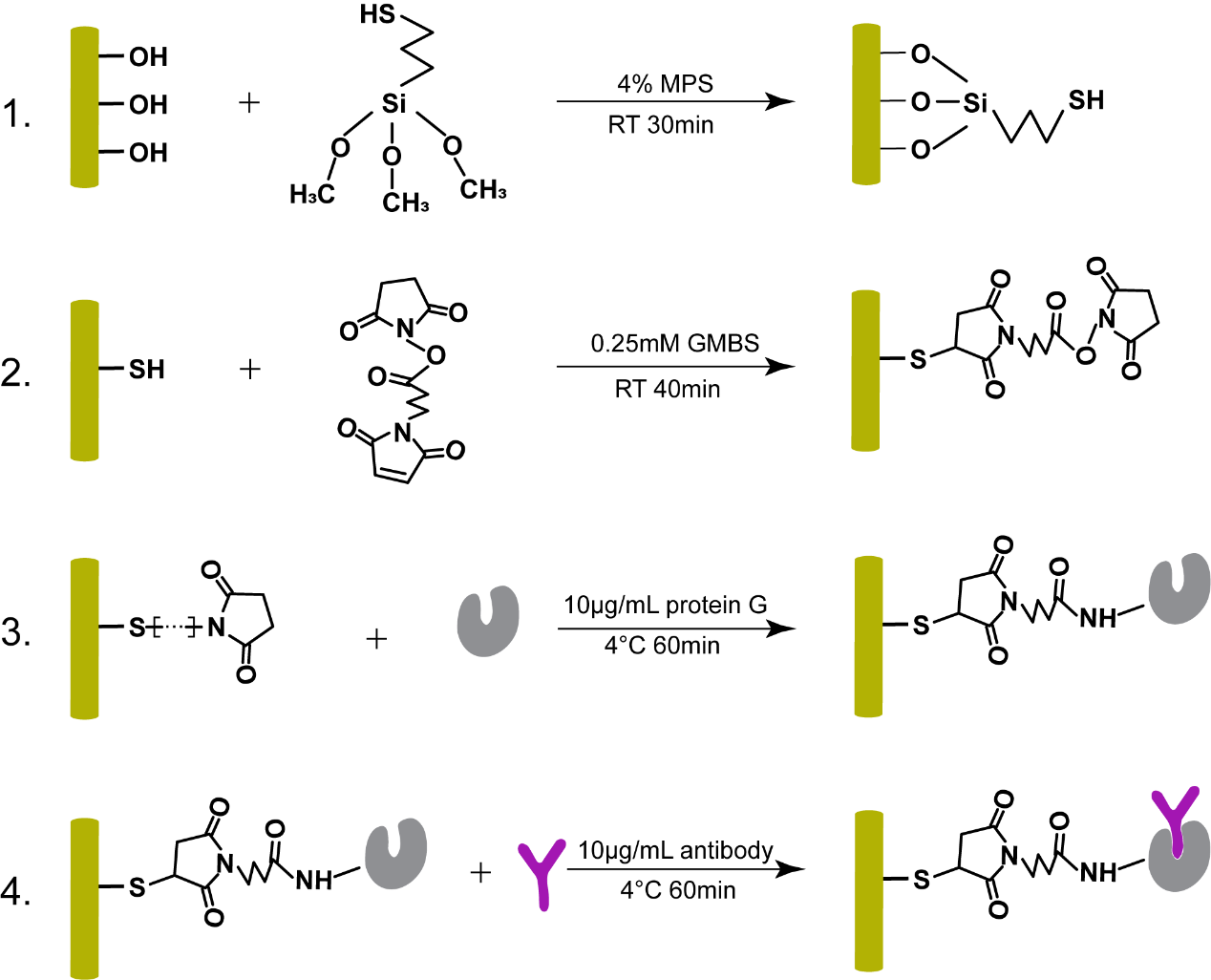
**

**Figure S1.** 1. Surface silanization of ZnO nanorods with (3-mercaptopropyl) trimethoxysilane (MPS). 2. Incorporation of N-maleimidobutyryl-oxysuccinimide ester (GMBS) onto the surface. 3. Immobilization of protein G onto the surface. 4. Conjugation of the antibody onto the surface.

S2. Fourier-transform infrared spectroscopy (FT-IR) spectra

**
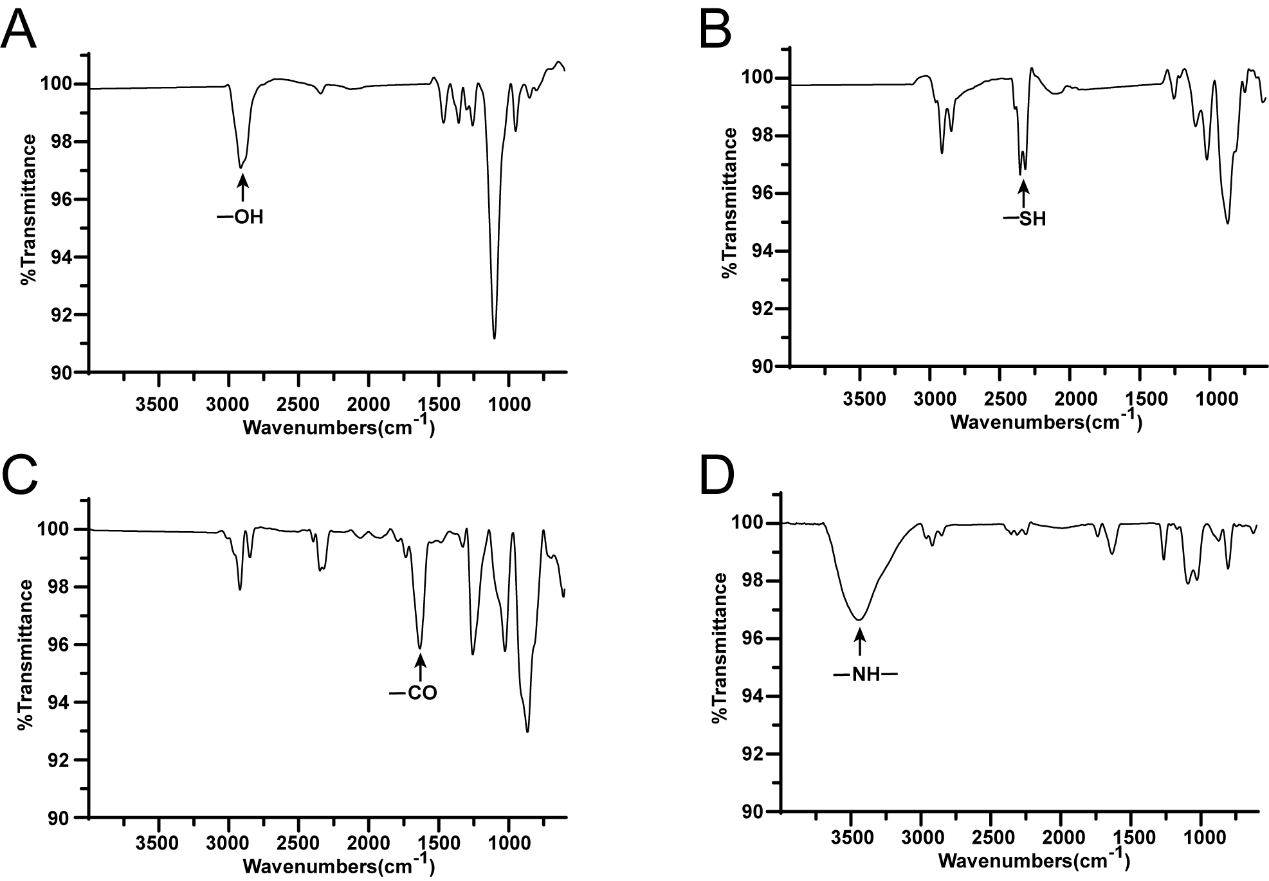
**

**Figure S2.** Fourier-transform infrared spectroscopy (FT-IR) spectra of each step in the process of ZnO nanorods covalent bond coupling to antibody.

**S3. Characterization of the antibodies on the surface of ZnO-nanorods**

**
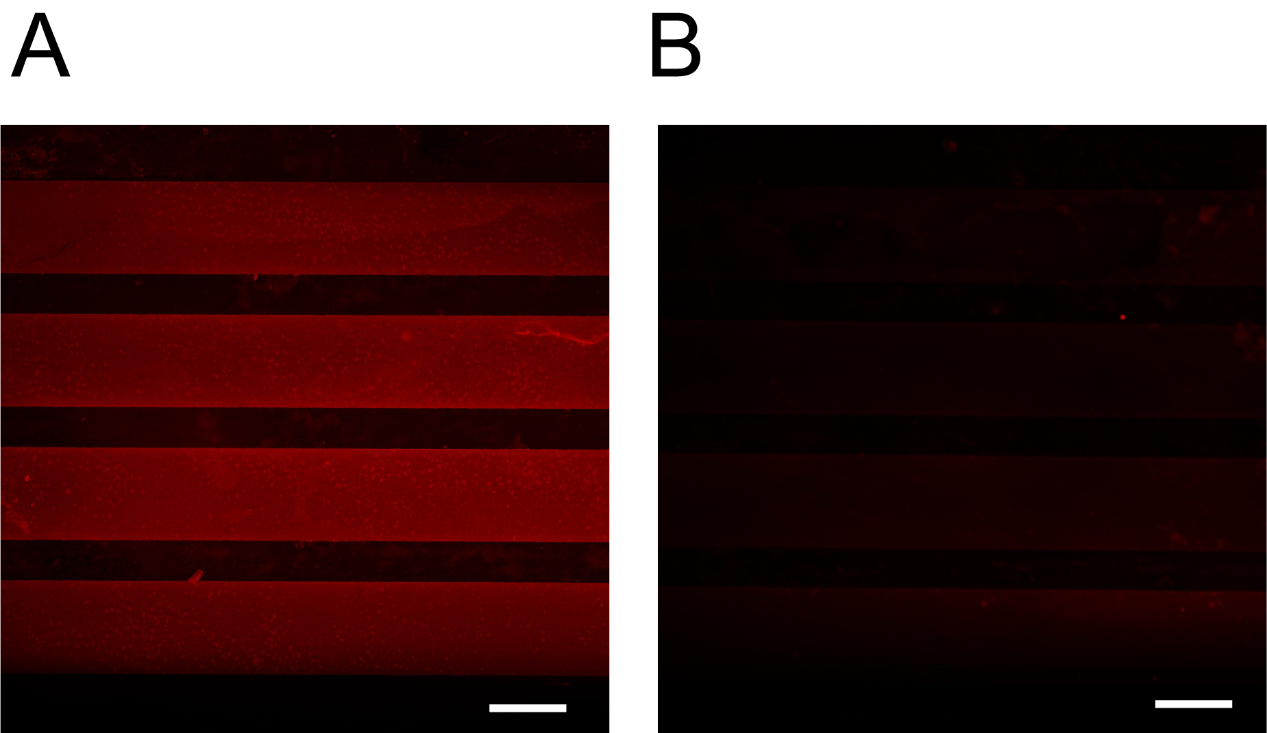
**

**Figure S3.** (A) Characterization of anti-CD63 and anti-CD81 antibody-functionalized ZnO-chip device by fluorophore-conjugated goat anti-mouse IgG. (B) Control. Scale bar: 200μm.

S4. Nanoparticle tracking analysis (NTA) of clinical samples

**
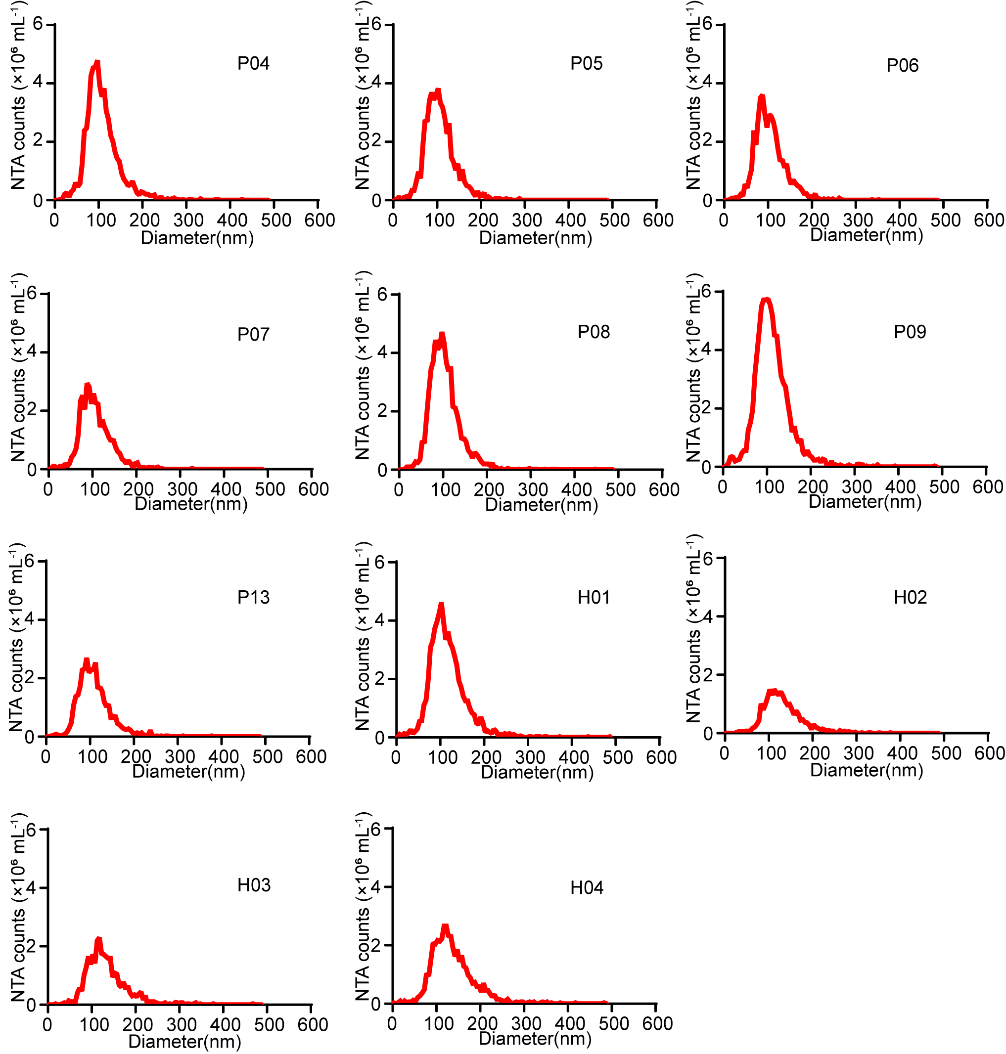
**

**Figure S4.** NTA data of 7 patients with osteosarcoma and 4 healthy donors.

S5. The co-fluorescence on the ZNI chip


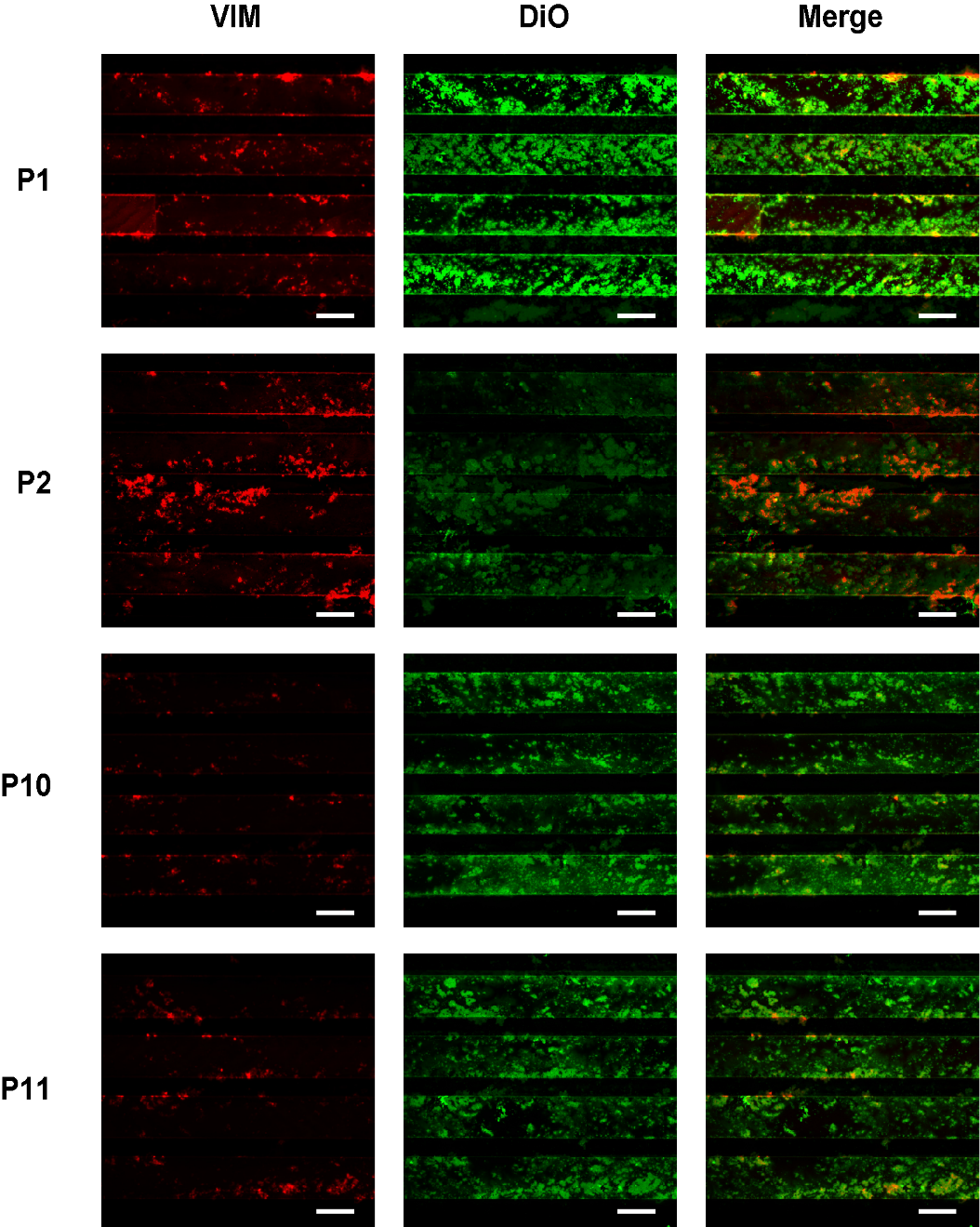


**Figure S5.** Co-fluorescence image of VIM (red) and DiO (green) from patient 1, patient 2, patient 10 and patient 11. Scale bar: 200μm.

**S6. Western blot analysis of extracellular vesicles from osteosarcoma cell line**

**
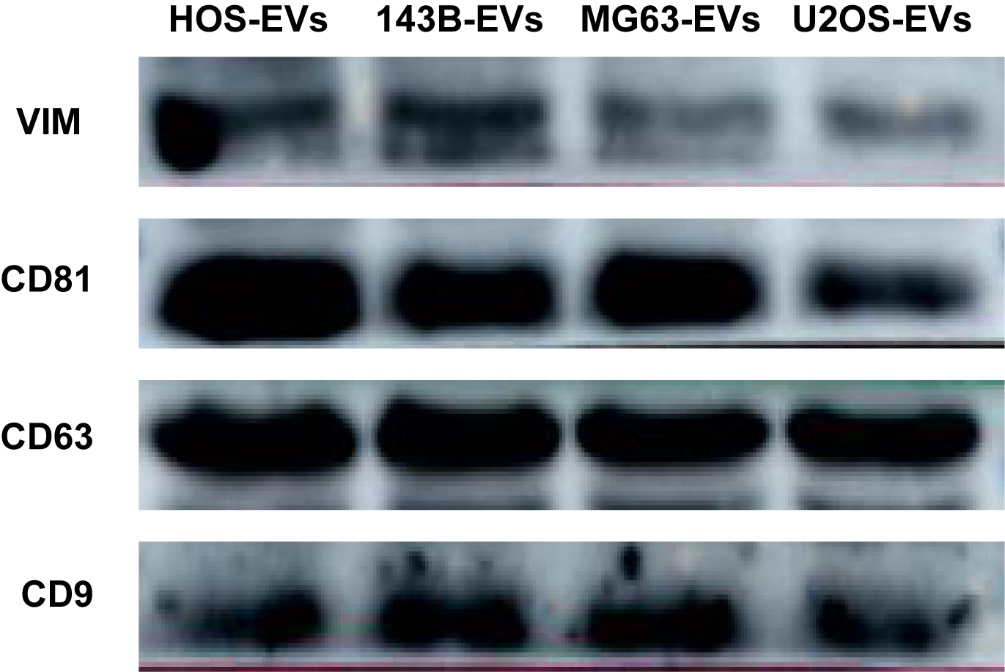
**

**Figure S6.** Western blot analysis of the protein markers of VIM, CD9, CD63 and CD81 in HOS, 143B, MG63 and U2OS extracellular vesicles (EVs).

**S7. Negative controls of DiO and VIM fluorescence
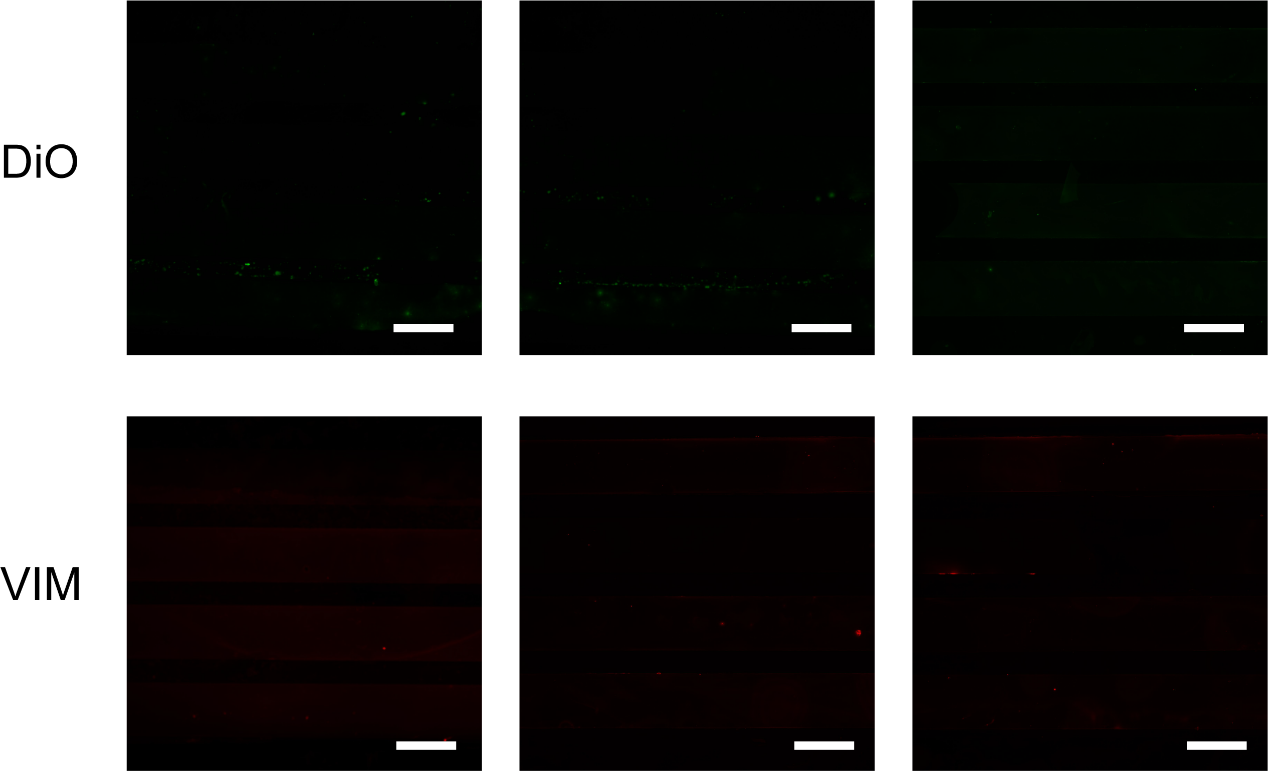
**

**Figure S7.** Fluorescence image of DiO (green) and VIM (red) for negative control. Scale bar: 200μm.

| Target | Vendor | Product No. |
| --- | --- | --- |
| Polydimethylsiloxane (PDMS, RTV615) | Momentive | 20BWFA001 |
| Zinc nitrate hexahydrate | Sinopharm Chemical Reagent | 80141318 |
| Zinc acetate | Sinopharm Chemical Reagent | 31001427 |
| Hexamethylenetetramine (ammonia) | Sinopharm Chemical Reagent | 10010818 |
| Ammonia | Sinopharm Chemical Reagent | 10002108 |
| Acetone | Sinopharm Chemical Reagent | 10000418 |
| Isopropyl alcohol | Sinopharm Chemical Reagent | 40064360 |
| (3-mercaptopropyl) trimethoxysilane (MPS) | Absin | abs42041038 |
| N-maleimidobutyryl-oxysuccinimide ester (GMBS) | BACHEM | Q-2775.0500 |
| 3,3'-dioctadecyloxacarbocyanine perchlorate (DiO) | AAT Bioquest | 22066 |
| Polyethyleneimine (PEI) | Sigma-Aldrich | 764604 |
| Protein G | Sciencell | SC-1002-01 |
| Anti-CD9 monoclonal antibody | Abcam | ab2215 |
| Anti-CD63 monoclonal antibody | Abcam | ab59479 |
| Anti-CD81 monoclonal antibody | Abcam | ab59477 |
| Alexa Fluor 647 labeled goat anti-rabbit IgG (H+L) | Abcam | ab150079 |
| Alexa Fluor 647 labeled goat anti-mouse IgG (H+L) | Abcam | ab150115 |
| Rabbit anti-vimentin monoclonal antibody | Cell Signaling Technology | 5741S |

**Table S1. The materials used in this research.**
